# Hutchinson’s sign of ophthalmic zoster

**DOI:** 10.1002/ccr3.2596

**Published:** 2019-12-11

**Authors:** Johanna Frary, Pelle Trier Petersen, Manan Pareek

**Affiliations:** ^1^ Department of Oncology North Zealand Hospital Hillerød Denmark; ^2^ Department of Pulmonary and Infectious Diseases North Zealand Hospital Hillerød Denmark; ^3^ Department of Cardiology North Zealand Hospital Hillerød Denmark

**Keywords:** herpes zoster ophthalmicus, herpesvirus 3, human, trigeminal nerve

## Abstract

A 77‐year‐old woman presented with ophthalmic zoster and nasal tip involvement, consistent with Hutchinson's sign. Ocular examination disclosed a swollen upper eyelid, chemosis, conjunctival injection, pus, and mild corneal endothelial decompensation. The presence of Hutchinson's sign requires urgent consultation with an ophthalmologist due to the high risk of ocular complications.

## Graphical abstract

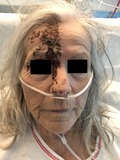

## 1 CASE HISTORY

**Figure 1:**
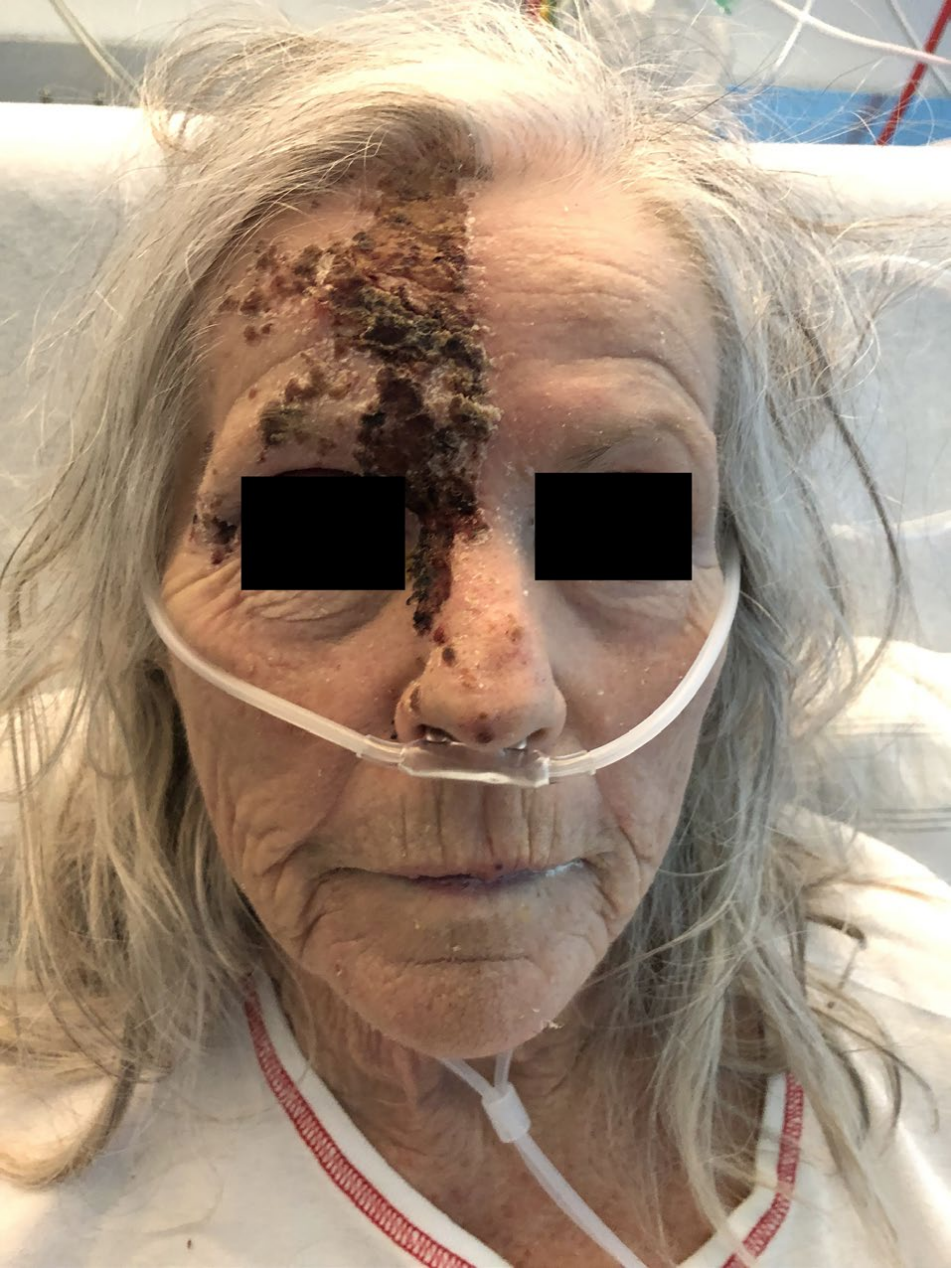
Multiple crusted vesicles in the distribution of the right ophthalmic nerve, that is, the forehead, nasal bridge, and the nasal tip, consistent with Hutchinson's sign of ophthalmic zoster

**Figure 2:**
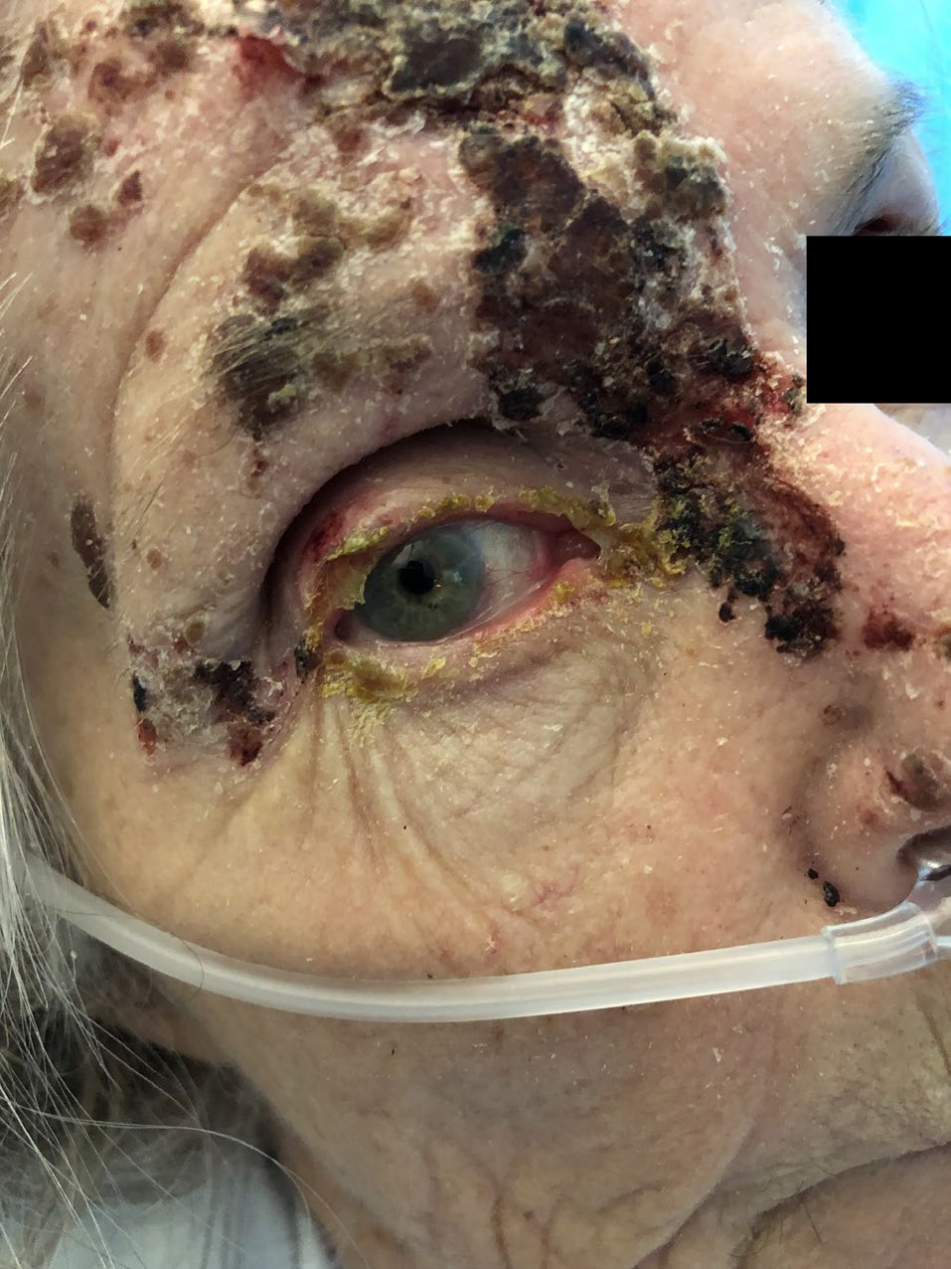
Ocular affection in ophthalmic zoster

A 77‐year‐old woman (Table 1) presented with a 6‐day history of right‐sided facial pain, blurred vision, and photosensitivity. Physical examination showed crusted vesicles in the distribution of the right ophthalmic nerve (Figure 1). Findings were consistent with Hutchinson's sign of ophthalmic zoster. Ocular (including slit lamp and fundus) examination disclosed a swollen upper eyelid, chemosis, conjunctival injection, pus, and mild corneal endothelial decompensation (Figure 2). Visual acuity was limited to hand motion. There were no cells or flare in the anterior chamber, although the examination was complicated by the swollen eyelid. Oral acyclovir therapy was begun. Four days later, the patient was admitted due to confusion and malaise. Chest X‐ray showed a right‐sided pulmonary infiltrate. Staphylococcus aureus superinfection was identified in her zoster lesions. Although intravenous acyclovir as well as antibiotic and supportive therapy was initiated, the patient died due to respiratory complications.

**Table 1:** Past medical history and list of medications at admission

Past medical history
Intracranial hemorrhage Ischemic stroke Gout Heart failure with reduced ejection fraction (left ventricular ejection fraction 25%‐30%) Stage 4 chronic kidney disease
List of medications
Allopurinol 100 mg once daily Cholecalciferol 35 μg once daily Clopidogrel 75 mg once daily Doxazosin 4 mg once daily Ferrous fumarate 66 mg twice daily Furosemide 40 mg once daily Lercanidipine 10 mg once daily Methyldopa 250 mg thrice daily Paracetamol 1000 mg as needed Simvastatin 40 mg once daily

Nasal tip, side, or root involvement during ophthalmic zoster represents the dermatome of the nasociliary nerve.^1^ Ocular involvement is more common in such cases.^2^ Accordingly, this requires urgent ophthalmological consultation. However, subsequent studies have found that even though the risk among these patients is much higher than those without, about a third of patients without nasociliary nerve involvement may also develop ocular complications.^3^

## CONFLICT OF INTEREST

None declared.

## AUTHOR CONTRIBUTIONS

All authors: participated in collecting patient data (pictures and clinical history), reviewing the literature, interpretation of clinical findings, drafting the manuscript, critical revision of the manuscript for important intellectual content, and approval of the final version.
